# P-258. A Longitudinal Study on the Impact of Sink Interventions on Environmental Bioburden and Patient Infections within Two Intensive Care Units at an Academic Hospital

**DOI:** 10.1093/ofid/ofae631.462

**Published:** 2025-01-29

**Authors:** Katie Gravagna, Emily Sickbert-Bennett, Sharon Thompson, Bobby G Warren, Cyndi Culbreth, Kate Schultz, Kevin Alby, Aaron Barrett, Amanda M Graves, Lauren DiBiase, Guerbine Fils-Aime, Becky A Smith, Deverick J Anderson, David J Weber

**Affiliations:** University of North Carolina at Chapel Hill, Chapel Hill, North Carolina; UNC Medical Center, Chapel Hill, North Carolina; UNC Medical Center, Chapel Hill, North Carolina; Duke University School of Medicine, Hillsborough, North Carolina; UNC Healthcare, Chapel Hill, North Carolina; UNC Hospitals, Chapel Hill, NC; University of North Carolina, Chapel Hill, North Carolina; Duke Health, Cary, North Carolina; Duke University School of Medicine Duke Center for Antimicrobial Stewardship and Infection Prevention, Durham, North Carolina; UNC Health Care, Chapel Hill, NC; Duke School of Medicine, Durham, North Carolina; Duke University, durham, North Carolina; Duke Center for Antimicrobial Stewardship and Infection Prevention, Durham, NC; University of North Carolina School of Medicine, Chapel Hill, NC

## Abstract

**Background:**

Patient room sinks are frequently identified as potential sources for hospital-associated infections (HAIs), but there are no clear recommendations for interventions to reduce sink-related pathogenic bioburden. We conducted one of the first longitudinal crossover trials to assess the impact of practical sink interventions in an academic hospital.

**Figure 1.**
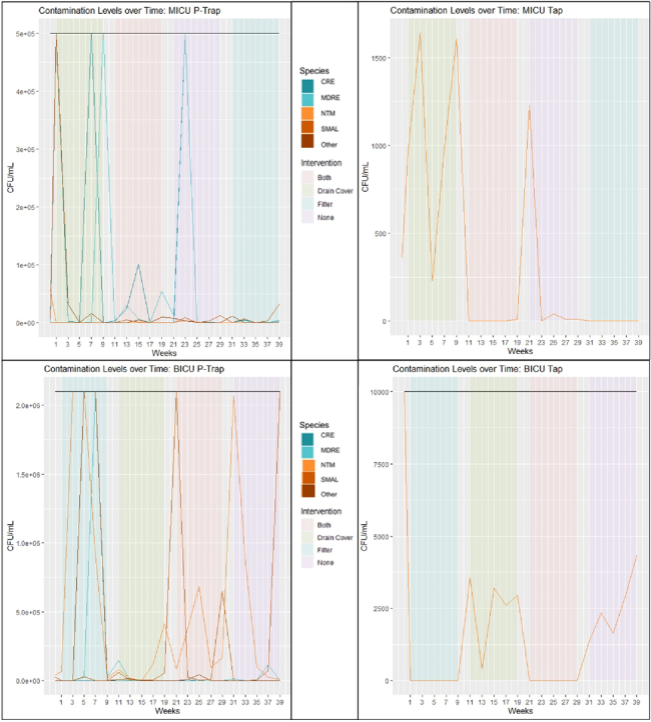
Contamination levels in patient room sink p-trap and tap water between units over time. Black lines represent samples with CFU/mL above the limit of detection.

**Methods:**

We conducted a 4-phase randomized crossover trial in a medical ICU (MICU) and burn ICU (BICU). Each phase was 8 weeks with a 2-week washout between phases: drain covers, point-of-use filters, both, or no intervention on patient room sinks by unit. Environmental samples from tap water, p-trap water, air, and swabs of the sink basin and adjacent surface were collected every 2 weeks from 5 patient room sinks per unit. Colony-forming units (CFU)/mL of non-tuberculous mycobacteria (NTM), carbapenem-resistant *Enterobacterales* (CRE), multidrug-resistant *Enterobacterales* (MDRE), and *Stenotrophomonas maltophilia* were calculated following MALDI-TOF speciation. Patient HAI results were also recorded.

**Results:**

Without filters, 68.7% (68/99) of tap water samples were positive, compared to 2.2% (2/90) with filters (p< 0.05). P-trap samples were highly variable with high bioburden and no clear time trends at the room or unit level (**Figure 1**). Positive air and surface samples rarely occurred but were most common in the drain cover phase (12/135). Within rooms, environmental samples were primarily positive before patient clinical isolates of the same species (7/8). Tap water samples across both units were primarily positive for NTM (71/189) yet no study-room-associated NTM infections were identified.

**Conclusion:**

We identified that sinks, particularly p-traps, present a consistent source of pathogenic bioburden with fluctuating populations yet rarely contributed to surface/air contamination with organisms of interest in ICU patient rooms. Neither intervention impacted p-trap colonization (CFU/mL). We found that point-of-use filters consistently and effectively removed NTM from tap water. Drain covers were not found to be a useful intervention for reducing surface bioburden. Future research on decolonizing pathogenic bacteria from patient room p-traps is needed.

**Disclosures:**

**Becky A. Smith, MD**, UpToDate: royalties

